# Sequencing accuracy and systematic errors of nanopore direct RNA sequencing

**DOI:** 10.1186/s12864-024-10440-w

**Published:** 2024-05-28

**Authors:** Wang Liu-Wei, Wiep van der Toorn, Patrick Bohn, Martin Hölzer, Redmond P. Smyth, Max von Kleist

**Affiliations:** 1 Systems Medicine of Infectious Disease (P5), Robert Koch Institute, Berlin, Germany; 2 International Max-Planck Research School ‘Biology and Computation’, Max-Planck Institute for Molecular Genetics, Berlin, Germany; 3 Helmholtz Institute for RNA-based Infection Research, Helmholtz Centre for Infection Research, Würzburg, Germany; 4 Genome Competence Center (MF1), Robert Koch Institute, Berlin, Germany; 5 Faculty of Medicine, University of Würzburg, Würzburg, Germany; 6 Department of Mathematics and Computer Science, Freie Universität, Berlin, Germany

**Keywords:** Nanopore sequencing, Direct RNA sequencing, Transcriptomics, Epitranscriptomics, Sequencing errors, Sequencing accuracy, SQK-RNA004

## Abstract

**Background:**

Direct RNA sequencing (dRNA-seq) on the Oxford Nanopore Technologies (ONT) platforms can produce reads covering up to full-length gene transcripts, while containing decipherable information about RNA base modifications and poly-A tail lengths. Although many published studies have been expanding the potential of dRNA-seq, its sequencing accuracy and error patterns remain understudied.

**Results:**

We present the first comprehensive evaluation of sequencing accuracy and characterisation of systematic errors in dRNA-seq data from diverse organisms and synthetic in vitro transcribed RNAs. We found that for sequencing kits SQK-RNA001 and SQK-RNA002, the median read accuracy ranged from 87% to 92% across species, and deletions significantly outnumbered mismatches and insertions. Due to their high abundance in the transcriptome, heteropolymers and short homopolymers were the major contributors to the overall sequencing errors. We also observed systematic biases across all species at the levels of single nucleotides and motifs. In general, cytosine/uracil-rich regions were more likely to be erroneous than guanines and adenines. By examining raw signal data, we identified the underlying signal-level features potentially associated with the error patterns and their dependency on sequence contexts. While read quality scores can be used to approximate error rates at base and read levels, failure to detect DNA adapters may be a source of errors and data loss. By comparing distinct basecallers, we reason that some sequencing errors are attributable to signal insufficiency rather than algorithmic (basecalling) artefacts. Lastly, we generated dRNA-seq data using the latest SQK-RNA004 sequencing kit released at the end of 2023 and found that although the overall read accuracy increased, the systematic errors remain largely identical compared to the previous kits.

**Conclusions:**

As the first systematic investigation of dRNA-seq errors, this study offers a comprehensive overview of reproducible error patterns across diverse datasets, identifies potential signal-level insufficiency, and lays the foundation for error correction methods.

**Supplementary Information:**

The online version contains supplementary material available at 10.1186/s12864-024-10440-w.

## Background

Envisioned in the 1980s and developed over the next three decades, the nanopore sequencing technology has transformed the DNA and RNA sequencing landscape in recent years [1, 2]. Sequencing platforms released by the Oxford Nanopore Technologies (ONT) have enabled high-throughput long-read sequencing of single DNA/RNA molecules with low experimental requirements. Recently, it was reported that DNA reads from the latest R10.4 chemistry achieved state-of-the-art accuracy on bacterial genome assembly without short read polishing, thanks to significant improvements in the accuracy of homopolymer regions [3].

In addition to DNA sequencing, ONT offers currently the only commercial platform for the direct sequencing of RNA molecules (dRNA-seq) [4, 5]. Ligated to a DNA adapter containing a helicase motor, a polyadenylated RNA molecule is translocated in the 3’–5’ direction through a protein nanopore embedded in an electrically charged membrane. The translocation causes systematic disruptions to the ionic current flow, characteristic of the nucleotides passing through the pore at the time. The current signals are basecalled into nucleotide sequences with machine learning algorithms, commonly known as “basecallers”. Ideally, these basecallers are trained on diverse organisms for better generalization and fewer biases to rare sequences and species.

Traditionally, high-throughput RNA sequencing protocols have relied on reverse transcription and/or amplification, which introduce various errors and biases that confound downstream analyses [6, 7]. In comparison, nanopore dRNA-seq avoids these biases and at the same time, produces reads up to thousands of bases in length. The ability to cover full-length gene transcripts at single read resolution can significantly improve on analyses traditionally complex with short read RNA-seq, such as identifying transcript isoforms and quantifying Poly-A tail lengths [8–10]. So far, nanopore dRNA-seq has been applied to diverse organisms such as DNA and RNA viruses [11–14], bacteria [15], archaea [16], plants [17–19], yeast [20, 21], fish [22], mouse [23] and humans [8, 24].

Another promising application of dRNA-seq is the characterisation of the “epitranscriptome”. Although over 100 types of RNA modifications are known, only a small subset of them, such as m^6^A, m^5^C, and pseudouridine, are transcriptome-wide detectable [25], albeit with limited accuracy [26]. By sequencing RNA molecules directly, dRNA-seq has the potential to produce characteristic signals not only for the four canonical RNA bases but also for the diverse family of RNA base modifications. So far, several functionally important RNA modifications are found to be detectable with dRNA-seq, with a heavy focus on m^6^A [14, 19, 20, 24, 27], but also pseudouridine [28] and inosine [29]. These modifications are detected by computationally identifying systematic deviations in signal levels or basecalling errors at potentially modified positions. This is typically performed by including unmodified control samples (gene knockouts or in vitro transcribed RNA) as baseline, due to the abundance of background errors even in unmodified RNAs.

Despite the growing interest in this technology, nanopore dRNA-seq has been widely perceived as error-prone, with reported read accuracies of around 90% for single species [5, 15, 18], which hinders the downstream applications (by requiring control samples or short-read polishing) and limits its wider popularisation. A systematic examination and characterisation of dRNA-seq accuracy and error patterns could help 1) clarify its current limitations and potential, 2) direct future computational methods to account for the underlying uncertainty/errors, and 3) establish the foundation for improving pore chemistry and basecalling algorithms. Here, starting from the raw signal data, we re-analysed twelve public datasets covering a wide taxonomic range and present the first comprehensive benchmark of dRNA-seq accuracy using standardized metrics. Notably, systematic errors exist at both single base and motif levels that are reproducible across all investigated organisms, and such errors show strong and complex dependency on their local sequence contexts. In addition, we examined the relationship between read quality scores and error rates, and how adaptor detection failure can impact the read quality of short sequences. Lastly, following the release of the latest SQK-RNA004 sequencing kit at the end of 2023, we evaluated and compared its accuracy and error profiles to SQK-RNA002 using in-house datasets.

## Methods

### Data sources

We collected public dRNA-seq datasets covering a diverse range of organisms and in vitro transcribed RNAs, sequenced with the ONT direct RNA kits SQK-RNA001 and SQK-RNA002. Sequencing datasets in raw *fast5* files from the following studies are included in the analysis of sequencing accuracy and errors:

1. native and in vitro transcribed human data [21],
2. the house mouse *Mus musculus* [23],
3. yeast wildtype strain SK1 [20],
4. *Escherichia coli* [15],
5. in-cell and in vitro transcribed SARS-CoV-2 [13],
6. *Caenorhabditis elegans* [30],
7. the zebrafish *Danio rerio* [22],
8. *Arabidopsis thaliana* [17],
9. *Haloferax volcanii* [16],
10. in vitro transcribed short RNAs [28], from a) *Bacillus subtilis* guanine riboswitch, b) *B. subtilis* lysine riboswitch, and c) *Tetrahymena* ribozyme (reference length 202, 274 and 460 bases, respectively).

The reference sequences for organisms 1), 2), 4), 6), 7), 8), and 9) are obtained from the NCBI reference genome database and are respectively Human Build 38 GRCh38.p14, Mouse Build 39 GRCm39, *E. coli* ASM584v2, *C. elegans* WBcel235, Zebrafish Build 11 GRCz11, *A. thaliana* TAIR10.1 and *H. volcanii* DS2 ASM2568v1, while the references for 3), 5) and 10) are provided by the original authors on the respective GitHub repositories.

### Basecalling, mapping and filtering

Each dataset consists of raw *fast5* files that were basecalled with the ONT basecaller Guppy (version 6.1.7) using the RNA model rna_r9.4.1_70bps_hac, with the option --disable_qscore_filtering to switch off the default read Q-score filtering threshold of 7.

The basecalled reads in *fastq* format are aligned to the respective reference genomes with minimap2 [31] version 2.24, with the following options -ax splice -k14 -uf -secondary=no --eqx --sam-hit-only, which allows for split read alignment and disables secondary alignments. Aligned reads are further filtered to exclude supplementary alignments and reads with a mapping quality score (“MapQ”) lower than 20 or shorter than 100 bases.

To compare Guppy with the community-published basecaller RODAN [32], we used the pretrained model provided at its GitHub repository (https://github.com/biodlab/RODAN). As RODAN failed to process some of the older versions of single read *fast5* files for some datasets, we converted them into multi-read *fast5* files using the single_to_multi_fast5 function provided in the ont_fast5_api library (https://github.com/nanoporetech/ont_fast5_api).

### Definition of read accuracy

After alignment and filtering, the alignment errors (mismatches, insertions and deletions) in the reads are counted by parsing through the CIGAR string in the .sam file format. For evaluating read accuracy, we follow the definition in [33],

$$\begin{aligned} \text {Read Accuracy} = \frac{N_{match}}{N_{match} + N_{mis} + N_{del} + N_{ins}} \end{aligned}$$

where $N_{match}$ and $N_{mis}$ are the number of bases on a read that are reported as matches and mismatches, and $N_{del}$ and $N_{ins}$ are the total length of all deletions or insertions as reported by the aligner. This definition of read accuracy is also commonly known as the “BLAST identity” and has been used by ONT as the official metric of read accuracy (https://labs.epi2me.io/quality-scores/).

Similarly, the read-level mismatch, insertion and deletion rate is defined as

$$\begin{aligned} \text {Mis/Ins/Del} = \frac{N_{mis/ins/del}}{N_{match} + N_{mis} + N_{del} + N_{ins}} \end{aligned}$$

### Definition of base and motif errors

To study error patterns at single nucleotide and motif levels, we define the accuracy of a base or a motif in a dataset as the frequency of the base/motif being correctly basecalled/matched as the reference base/motif, e.g. a 90% accuracy of base A would be equivalent to A being correctly basecalled 90% of the times over the whole dataset. Similarly, a mismatch/deletion error rate of a base/motif is defined as the frequency of the base/motif being mismatched or deleted. In the case of motifs (length $\ge 2$), both partial and complete mismatches and deletions are counted as a single instance of error. On the other hand, an insertion error rate of a motif is defined as the frequency of the motif having inserted base(s) anywhere between its first and last base (independent of the length of insertion). For example, GAA and GAAA are both counted as an insertion error for the motif GA. For tractable computation, larger datasets are subsampled to 200,000 reads each and reads aligned to soft–masked positions (represented as lowercase letters) and ambiguous positions (represented as N/n) in the reference genomes are excluded.

For the comparative analysis of hetero- and homopolymers, we define homopolymers as motifs consisting of only one nucleotide type, starting from length 2. For each aligned read, the aligned segment of the reference sequence is parsed and positions of homopolymers are recorded. Homopolymers of a certain length are counted only once and not further counted as multiple instances of shorter motifs (e.g. AAAA is counted as one homopolymer motif of length 4 and not two of length 2). Heteropolymers are defined as positions that do not belong to homopolymers and therefore, can also be of length 1. For example, in the case of AAGCC, there are two homopolymers of length 2 and one heteropolymer of length 1. Deletion and mismatch errors are counted and grouped based on whether their positions are homopolymeric. As indel errors are most often called at the two ends of a homopolymer (as opposed to in the middle), insertions are excluded in the comparison due to the ambiguity regarding whether they originated from a homopolymer or a heteropolymer. The accuracy of a homopolymer motif is defined as the frequency of the homopolymer being correctly basecalled (both the nucleotide type and length need to be correct).

For the analysis of context-dependent errors, we computed the frequency of incorrect basecalls of the center base within a 3-mer context where the first and last bases were correct, in order to limit errors propagating through the neighboring positions.

### Signal analysis

The signal event data in the basecalled *fast5* files outputted by Guppy were extracted using the ont_fast5_api library. Guppy first determines the start signal position of basecalling (named first_sample_template). The raw signals are first segmented with a chunk size of ten observations each and then the transitions between nucleotides are determined by Guppy and stored in the “Move” table. Therefore, the length of raw signals of a read is roughly first_sample_template + 10 * the length of Move.

The mean signal intensities and standard deviations are computed from the signal segments assigned to a base at only the correctly basecalled positions (based on alignment). The dwell times are computed as the length of the signal segments assigned to a base (and thus are multiples of 10).

To plot the signals of a 3-mer motif, due to the differences in dwell times, a running window is applied to extract the mean signal intensity with a step size of length of signals divided by 10 (and thus resulting in ten signal samples for each position). Only the correctly basecalled 3-mers at all three positions are included.

### Read quality score

In addition to the per-base quality scores typically found in *fastq* files, Guppy also outputs a quality score per read, “read Q-score”, which is reported as mean_qscore_template in the sequencing_summary.txt file. Q-scores are typically used as a filter for poorly sequenced and basecalled reads. Guppy, by default, classifies reads with a Q-score lower than 7 into the failed folder, which is usually excluded in downstream analysis.

As the quality scores are calibrated to follow the expected error distribution of Phred quality scores [34], the expected error probability of a read is then

1 $$\begin{aligned} \text {P} = 10^{\frac{-\text {Q-score}}{10}} \end{aligned}$$

where P is essentially 1 - Expected Read Accuracy. Consequently, Guppy’s default read filter at a Q-score of 7 corresponds to an expected error rate of 20%, or equivalently, a read accuracy of 80%.

Alternatively, the read Q-score can be obtained from *fastq* files by calculating the mean per-base error rate, followed by a back conversion to Phred Q-score:

2 $$\begin{aligned} \text {Read Q-score} = -10\log _{10} \left[ \frac{1}{N} \sum 10^{-q_i/10}\right] \end{aligned}$$

where $q_i$ is the individual base quality for base *i* in a read of length *N*.

### Sequencing of the curlcake sequences

To facilitate efficient evaluation of basecalling errors across all possible 5-mer sequences we employed the so-called “curlcake” sequences previously designed by [20]. We performed PCR amplification with Primestar GXL (Takara Bio) from plasmids (Addgene #139340-139343) using primers GCCGGTAATACGACTCACTATAGG and TTTTTTTTTTCAGGAAACAGCTATGACCATG according to manufacturer’s instructions, followed by quality control on agarose gel and purification with 0.5 volumes Mag-Bind TotalPure NGS (Omega Bio-tek) SPRI beads. In vitro transcription of 200 fmol DNA template was performed in a 10 $\mu$l reaction containing 40 mM Tris pH 7.5, 18 mM MgCl_2_, 10 mM DTT, 1 mM spermidine, 5 mM NTPs, 40 U RNasin (Molox), 1 $\mu$l YIPP (NEB) and homemade T7 RNA polymerase for 3 h at 37 ^∘^C, followed by addition of 9 $\mu$l H_2_ O and 1 $\mu$l DNase I-XT (NEB) followed by incubation at 37 ^∘^C for 30 min. RNA was then purified with 1.6 volumes of Mag-Bind TotalPure NGS (Omega Bio-tek) SPRI beads and 2 𝜇g was poly-adenylated with *E. coli* Poly(A) Polymerase (NEB) according to manufacturer’s instructions, followed by another SPRI bead purification with 1.6 volumes.

For direct RNA library preparation the a 10 $\mu$l ligation reaction was prepared containing 200 ng of poly-adenylated RNA, 1 $\mu$l of 1.4 $\mu$M RTA adapter, 2 $\mu$l NEBNext Quick Ligation Buffer (NEB), 0.2 $\mu$l RNasin (Molex) and 1 $\mu$l T4 DNA Ligase high concentration (NEB), incubated at room temperature for 15 min before purification with 0.5 volumes of SPRI beads. The ligated RNA was eluted in 10 $\mu$l RNase-free H_2_ O before ligation of the RMX or RLA motor protein for RNA kits SQK-RNA002 and SQK-RNA004 respectively according to manufacturer’s instructions. The prepared libraries were loaded onto a MinION R9.4.1 (FLO-MIN106D) flow cell or Promethion RNA (FLO-PRO004RA) flow cell respectively and sequenced for 24 hours.

The fast5 and pod5 files were basecalled with the ONT basecaller Dorado version 0.4.3, using the “rna002_70bps_fast@v3” model for SQK-RNA002 and “rna004_130bps_sup@v3.0.1” for SQK-RNA004 respectively. The analysis on read and motif errors are identical to previous sections.

## Results

### Deletions outnumber mismatches and insertions

We downloaded publicly available dRNA-seq datasets in raw *fast5* format consisting of both native and in vitro transcribed (IVT) samples of diverse organisms. The raw signal data were then basecalled into reads with the ONT basecaller Guppy, without the default read quality filtering. The basecalled reads were aligned to the respective references and further filtered by alignment quality and a minimum length threshold of 100. The statistics regarding each dataset included in the evaluation are given in Supplementary Table 1. The datasets range from 22,315 aligned reads in the archaeon *H. volcanii* to over 2.3 million aligned reads in the in vitro transcribed SARS-CoV-2 sample. For most organisms, the median read accuracy is within 88%–92%, with the mouse and the zebrafish datasets being the least accurate at 87.8% and 86.7%, respectively. The median read accuracy of $\sim$90% is in general agreement with previous reports for native transcriptomes of single organisms with dRNA-seq [5, 15, 18].

**Fig. 1:**
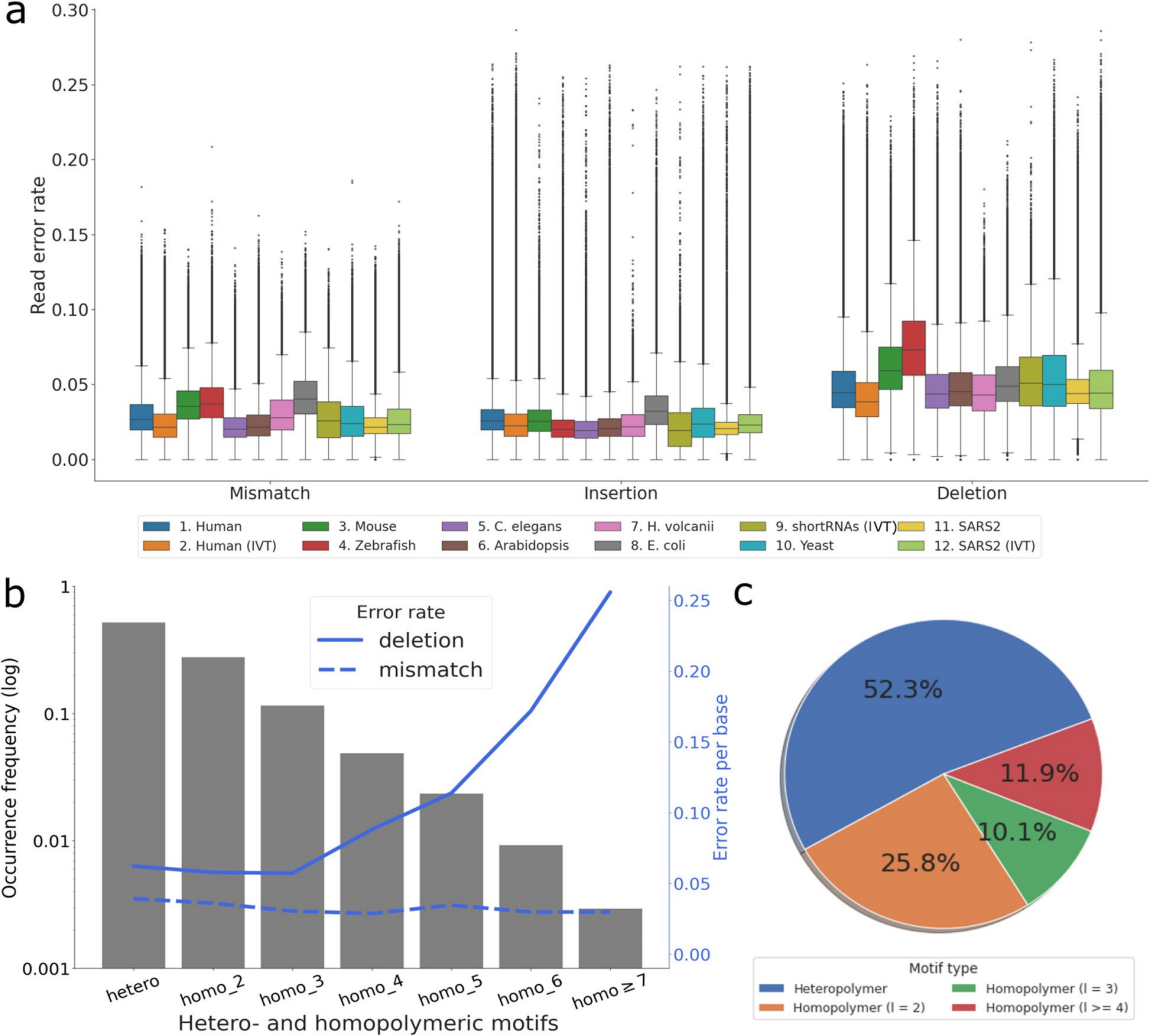
Sequencing accuracy of nanopore dRNA-seq and the error distribution in homopolymers versus heteropolymeric regions. **a** An overview of read error rate across diverse organisms, grouped by mismatch, insertion and deletion rate per read. Reads with accuracy lower than 70% were filtered for visualization. IVT – in vitro transcribed RNA. Samples are numbered from left to right in the figure legend. **b** The occurrence frequency distribution (left axis, in log scale) and the the error rate per base (right axis) in heteropolymeric and homopolymeric motifs of different lengths in the native human dataset. Error types are indicated using line styles. **c** The relative distribution of all mismatch and deletion errors in homopolymeric and heteropolymeric regions in the native human dataset. Homopolymer lengths are indicated in brackets and those of length longer than 3 are grouped together for visualisation

The distributions of mismatches, insertions and deletions are similar across most organisms, where deletions represent the most frequent type of error at around 5% per read, while mismatches and insertions each account for 2–3% (Fig. 1a, Supplementary Table 1). The reads from the IVT human sample have overall 1.5% fewer errors than the native human sample, possibly due to the absence of RNA base modifications. However, such a difference is not seen between the two SARS-CoV-2 samples.

To investigate whether the lengths of reads are related to their accuracy, we grouped reads by length, and found that the median read accuracy remains roughly constant as read length increases (Supplementary Figure 1a, b). However, the distribution of read accuracy tends to have a larger variance in shorter reads.

### Homopolymers and heteropolymers contribute equally to sequencing errors

While homopolymeric regions (motifs consisting of repeats of one nucleotide) represent the majority of errors in nanopore DNA sequencing [3, 35, 36], heteropolymers and short homopolymers contribute similarly to the overall sequencing errors in dRNA-seq data. In the native human transcriptome, heteropolymers and short homopolymers (of length 2 and 3) represent 91.5% of the sequenced dataset (Fig. 1b, left axis) and exhibit similar deletion and mismatch rates (Fig. 1b, right axis). Although longer homopolymers are more errorneous with higher deletion rate, they are also much rarer (less than 1% of nucleotides are in homopolymers over length of 7). The relative abundance of hetero- and homopolymers result in more than 50% of the deletion and mismatch errors arising from heteropolymeric regions (Fig. 1c, native human data) and is similar across datasets of other species (Supplementary Figure 2).

As expected, we observed an increase in the deletion rate of homopolymer motifs as the motif length increases, whereas mismatches remained stable (Fig. 1b, right axis). We then computed the accuracy of homopolymers of specific nucleotides at different lengths across the datasets and found that A/U homopolymers are more accurate than C/G homopolymers (Supplementary Figure 1c). Interestingly, in nanopore DNA sequencing datasets [36], higher accuracies of A and T homopolymer motifs have also been observed. This is likely due to the larger presence of A/U homopolymers in the training data for basecallers arising from their overrepresentation in genomes and transcriptomes [37]. Indeed, long A/U homopolymers are more abundant compared to C/G homopolymers across all the dRNA-seq datasets (Supplementary Figure 1d).

### Systematic errors of single nucleotides and motifs

**Fig. 2:**
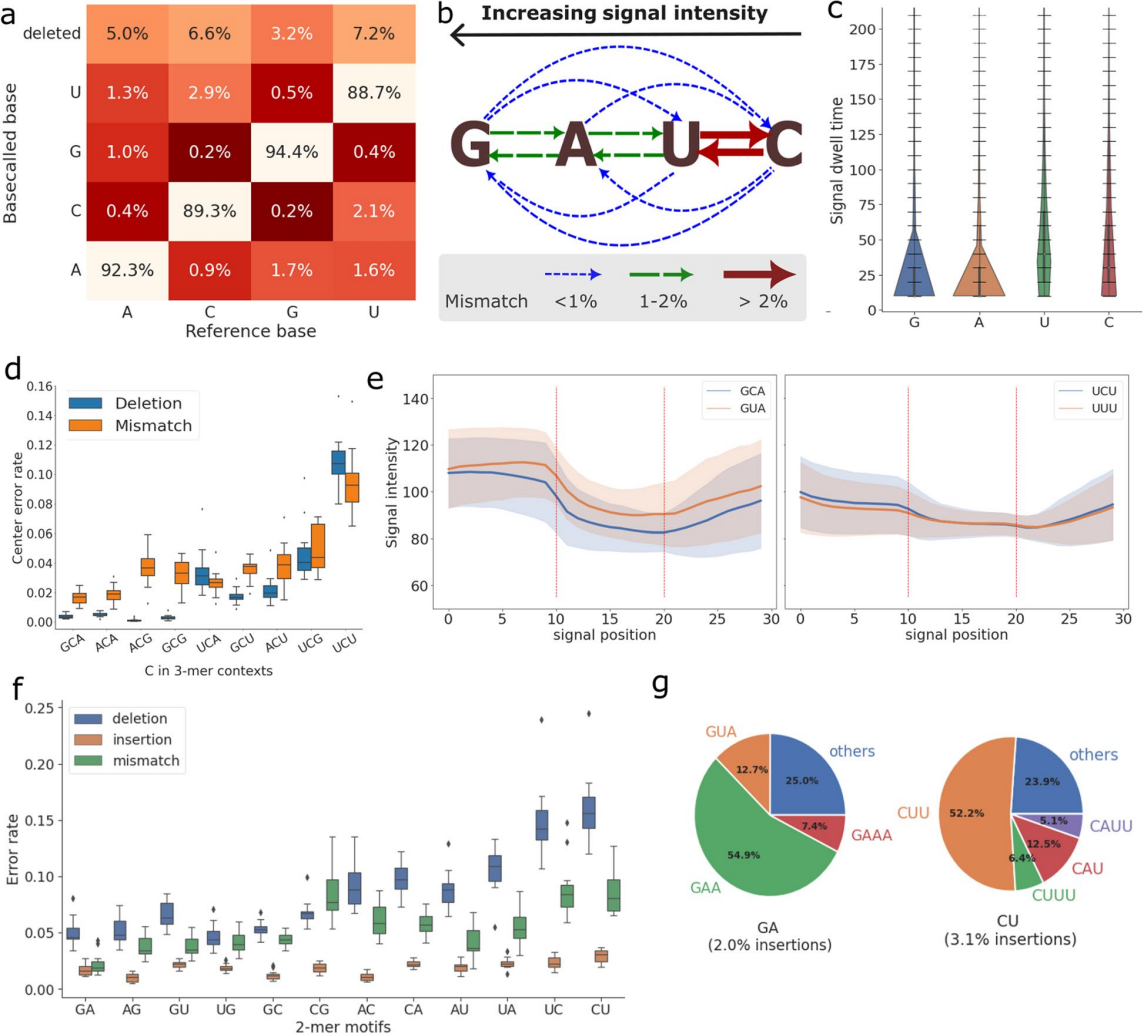
Systematic errors and context dependency in nanopore dRNA-seq. **a** Confusion matrix showing the frequencies of each base being correctly basecalled, miscalled or deleted, computed by taking the mean of all samples. **b** The relationship between signal similarity of nucleotides and their mismatch profiles. The arrow types indicate the mismatch rate from one nucleotide to another. **c** The distributions of dwell time at correctly basecalled positions, grouped by nucleotide type. **d** The error profiles of C across heteropolymeric 3-mer contexts, conditional on that the neighboring two bases are correctly basecalled. 3-mers with internal homopolymeric motifs were excluded in the plot, due to ambiguity of deleted positions (for example, a C deletion of CCA could be assigned to the first or the center position). Each data point represents the mean error rate of all such 3-mer motifs in one dataset. **e** The signal distribution of two pairs of motifs (GCA vs. GUA and UCU vs. UUU) at correctly basecalled positions. The concrete lines are the mean signal intensity and the shaded error bars are the mean signal standard deviation at each position. As the dwell times can differ, a running window is applied to extract the mean signal intensity with a step size of the signal length divided by 10, resulting in 10 signal samples for each base. The red dotted lines represent the nucleotide boundaries at signal positions 10 and 20. **f** The error rate of 2-mer heteropolymeric motifs across the samples, grouped by error type. Each data point corresponds to the accuracy of a motif in one dataset. **g** The insertion error profiles of GA and CU motifs, based on the native human dataset. Inserted motifs fewer than 5% of the total number of insertions are grouped into “others”

To explore how sequencing errors are distributed at single nucleotide level, we computed the frequency of (in)correct basecalls across the four RNA bases (Fig. 2a, Supplementary Figure 3). Across all datasets, guanines have consistently the highest accuracy, whereas cytosines and uracils tend to be the least accurate. Similar to the accuracy at read level, deletions remain the most frequent type of error at the individual base level, but are much more prevalent in Cs and Us. In terms of mismatches, C bases are more likely to be basecalled as U while G bases are more often confused with A. The mismatch error patterns are consistent with the similarity of the raw signals between the bases (Fig. 2b), where the four bases ranked by signal intensity are G > A > U > C (Supplementary Figure 4a, left panel, thus C is closest to U). Among the signal features, the dwell times (the number of raw signal observations assigned to a single basecall by Guppy) of G and A bases are considerably shorter than U and C bases (Fig. 2c). The difference in dwell times could be possibly related to the larger amount of deletion errors of U and C bases, for which the basecaller may require more information to correctly determine the number of bases.

At each time step of nanopore sequencing, around five consecutive RNA bases reside in the pore simultaneously and produce a signal event, which, if distinct enough from the surrounding signals, will lead to a correct basecall. Consequently, the surrounding sequence neighborhoods of a particular position, also known as the sequence contexts, are known to have an effect on accuracy. To examine the impact of sequence contexts on the accuracy in dRNA-seq data, we extended the analysis of single nucleotide errors by including the 3-mer sequence contexts. Here we study the effect of sequence contexts by considering the mismatch and deletion rates of the center base in a 3-mer, in which the first and last bases are correctly basecalled. The strongest bias towards specific sequence contexts are found in cytosine (Fig. 2d), whereas guanine is the most robust as a center base over its sequence contexts (Supplementary Figure 4b). We did not observe a general correlation between mismatch and deletion errors, reaffirming that the two errors likely arise from different signal-level causes. Extending the sequence contexts from 3-mers to 5-mers further shows more detailed separation of error profiles (Supplementary Figure 5).

To exemplify the signal impact of sequence contexts, we analysed the raw signals of two 3-mer motifs surrounding cytosine, GCA and UCU, which are respectively the most and least accurate 3-mer centered at C, together with and their closest mismatched counterparts, GUA and UUU (Fig. 2e). While GCA and GUA motifs show more distinct separation in signal space at all three positions, the signal differences of UCU and UUU are minimal, especially at the center position. In such an absence of distinct signals, we suspect that the basecaller would have to rely on long-range information to determine the identity of the center nucleotide, resulting in increased complexity and errors.

Similarly, we found large differences between the errors of length-2 heteropolymeric motifs, especially for deletions and mismatches (Fig. 2f). Among the 2-mers, GA/AG are the most accurate while UC/CU have consistently the lowest accuracy. Interestingly, although most 2-mers are close to their mirrored counterparts (“XY” and “YX”), there exist clear differences between certain pairs. In particular, GC motifs have consistently higher accuracy than CGs. Whilst deletions were generally more common than mismatches and insertions, for some 2-mers mismatch errors were very close to deletions, or even higher as seen in the “CG” motif. One possible explanation for the lower deletion error in some 2-mers is that these motifs are more different in signal space, and as a consequence more distinguishable by the basecaller. Insertion errors were similar for all 2-mers, suggesting that insertions likely arise from signal noise that is not motif-specific. The inserted bases generally consist of repeats of the second base in the motif, but can also occasionally involve other bases, for example, GA $\longrightarrow$ GUA (Fig. 2g, Supplementary Figure 6).

### Estimating read errors from read and base quality scores

The basecaller-generated quality scores are a helpful estimate for read accuracy without relying on the availability of reference genomes. There are two types of quality scores provided by ONT basecallers such as Guppy, 1) read-level quality scores (Q-scores) in the sequence_summary.txt file, and 2) base-level quality scores encoded as ASCII characters found in *fastq* files for each base on each read. Both types of quality scores are Phred quality scores, e.g. Q10 is equivalent to an error rate of 10% and Q20 equivalent to 1% (Equation 1). For dRNA-seq basecalling with Guppy, the default read Q-score filter is set to 7 (corresponding to an expected read accuracy of 80%). Reads with a lower Q-score than 7 are stored in the *failed* folder and are typically not included in downstream analysis.

**Fig. 3:**
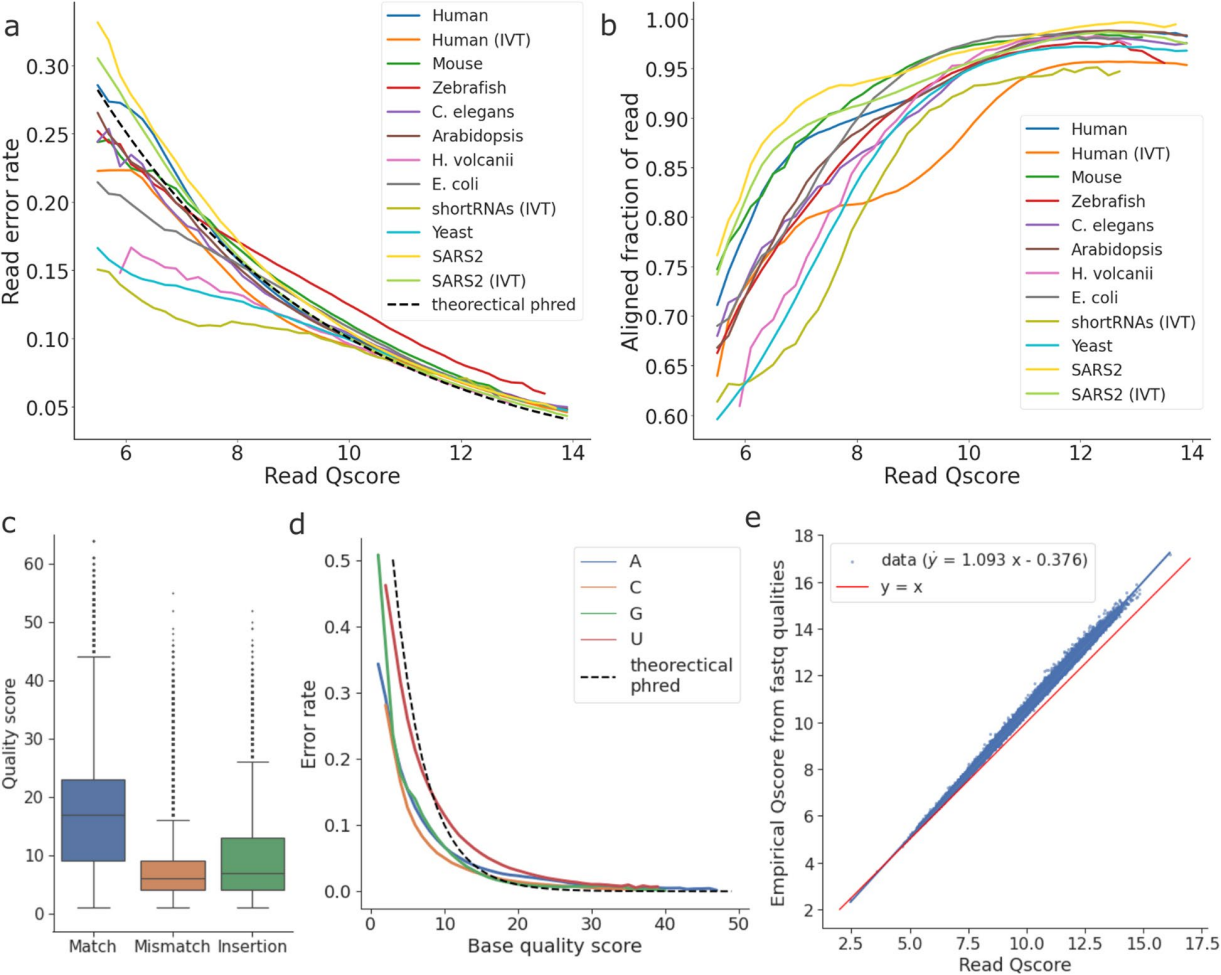
Read and base quality scores in dRNA-seq. **a** The relationship between read Q-scores and read error rates. First, Q-scores of each sample are divided into intervals of size 0.2 and the mean error rates of all reads belonging to their respective Q-score intervals are computed. The lines are then plotted by interpolating from the mean error rates of neighboring intervals. The dashed line is computed from Equation 1 and reflects the theoretical (expected) error rates based on the Phred quality scores. **b** The relationship between read Q-scores and the aligned fraction of reads after alignment for each dataset. **c** The distribution of per base quality scores, grouped by the per-base error type. **d** The relationship between per base quality scores and per-base error rate (mismatch rate + insertion rate), grouped by nucleotide. **e** The relationship between read Q-scores and “empirical” Q-scores computed from per base quality scores of reads in *fastq* files, based on the native human sample. The linear model is fitted by the ordinary least square method

To evaluate the accuracy of basecaller-estimated error rates, we compared the estimated error rate from read Q-scores with the empirical read error rates measured after alignment. For most organisms, the measured read error rates can be reasonably estimated at read Q-scores higher than 10, while at lower Q-scores the read error is often overestimated (Fig. 3a). This suggests that the default Q-score filter of 7 may be overly stringent. One important note here is that the measured read errors are a result of both basecalling and alignment. That is, aligners such as minimap2 employ “soft clipping” and do not always align reads at full length. Therefore, the empirical read error rates only reflect the errors at the aligned part of the reads. Indeed, reads with lower Q-scores tend to have more soft-clipped bases (Fig. 3b), indicating a larger number of errors at the start or end of the reads.

In addition to the read Q-scores, the base-level quality scores for each basecalled read can be useful in predicting per base errors in the basecalls (mismatches and insertions). We found that correctly sequenced bases tend to have higher quality scores than mismatched and inserted positions (Fig. 3c). Moreover, except for uracil, the per-base error rate can be accurately estimated when base-level quality scores are higher than 15 (Fig. 3d).

Theoretically, the read Q-scores are obtained by the log transformation of the mean per base error estimate, based on the relationship described in Equation 2. However, we observed a systematic deviation between the empirically computed Q-scores and the original read Q-scores reported by Guppy, especially at higher values (Fig. 3e). This deviation is found across all datasets and is highly linear (fitted with an ordinary least square model, p-value $\approx 0$).

### Adapter detection failure

**Fig. 4:**
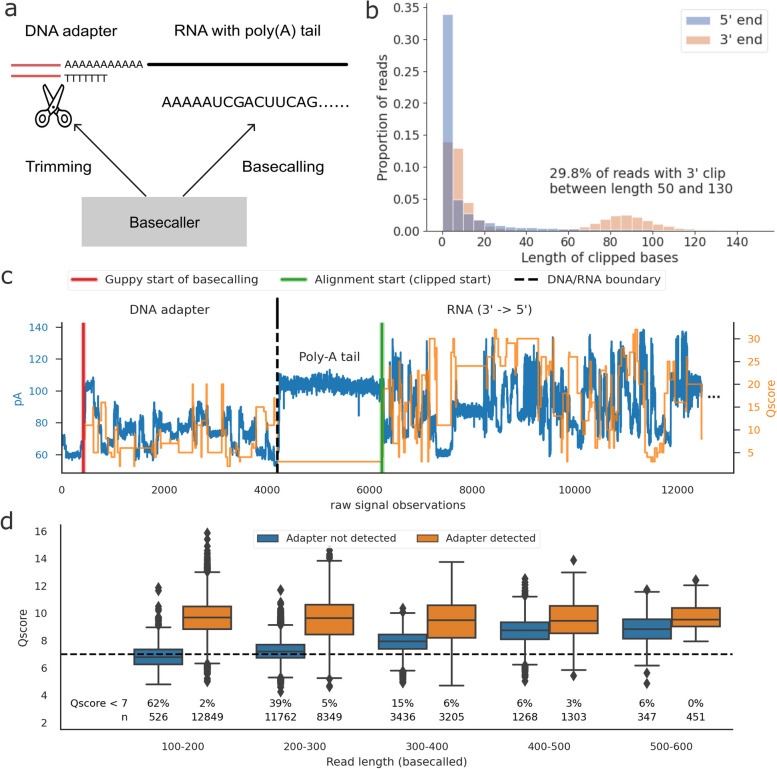
Adapter detection failure in Guppy. **a** A graphical illustration of the adapter trimming process. Polyadenylated RNA molecules are ligated with a DNA adapter at the 3’ end. The ONT basecaller will first perform adapter trimming before starting to basecall. **b** The length distribution of the soft-clipped bases at the 5’ and 3’ ends of reads, the latter of which shows a clear bimodal distribution, indicative of adapter detection failure, based on the native human sample. **c** A signal visualization of a selected read whose adapter failed to be detected by Guppy. The left y-axis shows the signal intensity value (in pA) and the right y-axis shows the quality scores per base. **d** The distribution of read Q-scores depending on whether the adapter was detected successfully, grouped by read length, based on the short RNA dataset. Adapter detection failure can reduce Q-score for short reads, leading to them being filtered at the conventional Q-score threshold of 7 (dashed line)

In dRNA-seq, RNA molecules are ligated to a 3’ DNA sequencing adapter (Fig. 4a). As a result, the start of the read contains signals from the DNA adapter. ONT basecallers such as Guppy have a built-in functionality to detect the end of the DNA adapter and begin basecalling only at the start of the RNA molecule (recorded as first_sample_template in the *fast5* files).

Whilst read lengths were not found to impact read accuracy (Supplementary Figure 1b), we observed systematic biases in the length distribution of soft-clipped bases. In contrast to the 5’ end, the length distribution of the soft-clipped bases at the 3’ end showed a clear bimodal pattern, with a second mode locating roughly between 50 and 130 bases and accounting for 30% of the reads (Fig. 4b, native human). The unique bimodality of 3’ soft-clips are found across datasets, with the second mode accounting for up to 41.5% of the reads in the zebrafish sample (Supplementary Figure 7), indicating that this is a common pattern in dRNA-seq data.

Upon manually inspecting the signal data of the reads, we discovered that the soft–clipped bases at the 3’ end were erroneous basecalls originating from the DNA adapter. For many of these reads, Guppy began basecalling at the beginning of the adapter, instead of at the start of the RNA segment. The beginning of reads with this issue typically contains a jump in signal intensity that is comparable to the one typically seen at the end of the adapter, marking the DNA/RNA boundary (Fig. 4c).

To evaluate the impact of adapter detection failure on the quality of downstream RNA basecalls, we compared the alignment error rates and basecall quality scores of reads with and without successful adapter detection. We found that basecalls in the DNA adapter are of low quality (Supplementary Figure 8a), but we did not observe that having these spurious basecalls at the start of the read negatively impacts the error rate of the subsequent RNA basecalls (Supplementary Figure 8b). However, for shorter reads (less than 400 bases), we observed that the spurious DNA basecalls significantly impact the read Q-score, dragging it below the default Q-score cutoff of 7 and leading to unnecessary data loss (Fig. 4d, Supplementary Figure 8c).

### Comparison with the community basecaller RODAN

For dRNA-seq data, RODAN [32] is currently the only non-ONT basecaller published by the research community. To examine whether RODAN has significantly improved over Guppy in basecalling performance, we re-basecalled all the datasets in this study with RODAN and compared its performance with Guppy (Supplementary Table 2). Among the datasets, four organisms (native human, *C. elegans*, *E. coli*, and *Arabidopsis*) were included in the training data of RODAN. RODAN performed 2–5% better than Guppy in three of these four datasets (except *Arabidopsis*). In the species outside its training set, RODAN has a 1–2% higher median read accuracy than Guppy on the mouse, *H. volcanii*, the short RNAs and the IVT SARS2 datasets; however, on the zebrafish dataset its median read accuracy is 4.8% lower than Guppy.

**Fig. 5:**
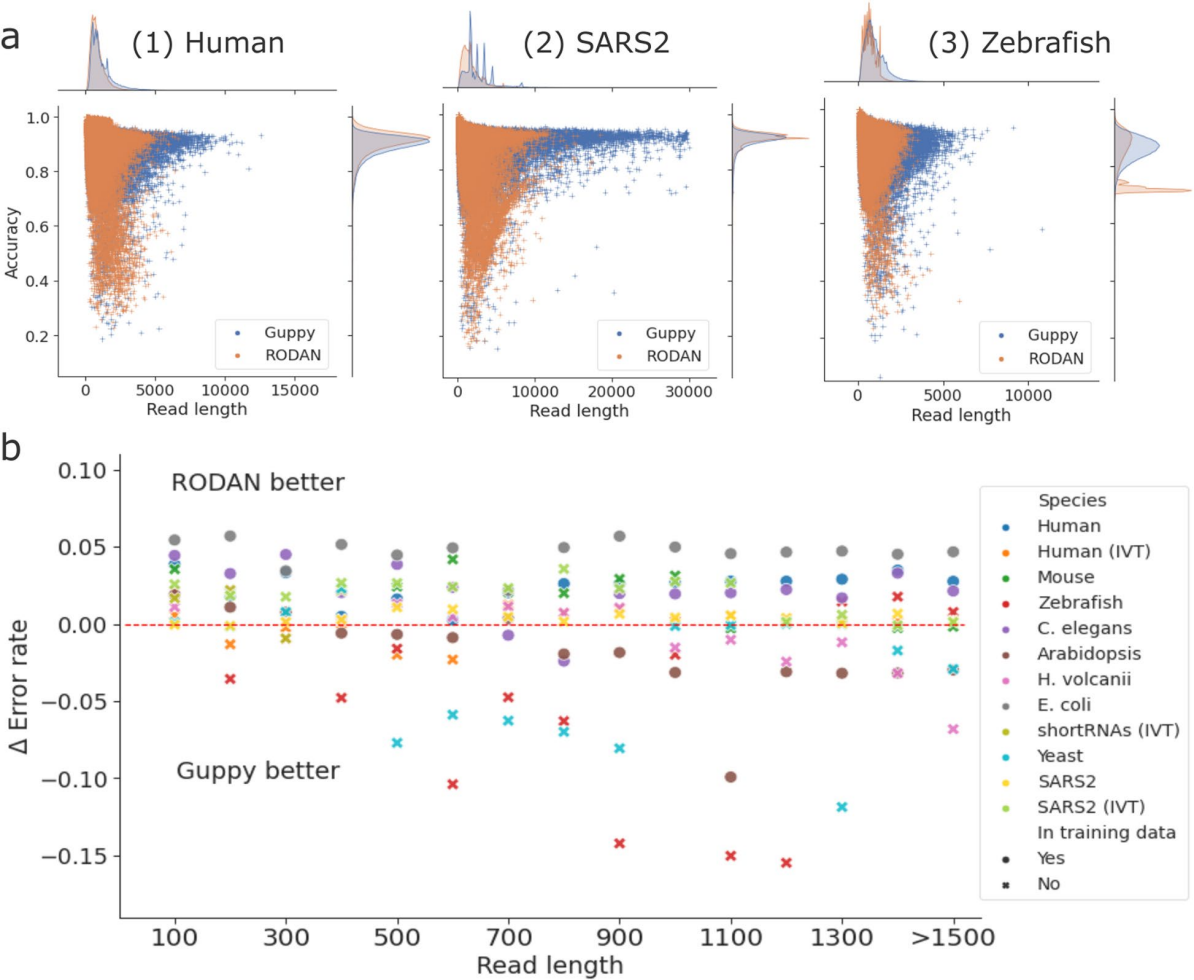
Comparison of basecalling performance between Guppy and the community basecaller RODAN. **a** The joint and marginal distributions of read length and read accuracy in 1) the native human, 2) SARS2 and 3) zebrafish samples. Each dataset was subsampled to 200,000 reads due to the runtime for kernel density estimation. **b** The relationship between aligned read length and the performance difference of RODAN versus Guppy. The $\Delta$ Error rate is Error_Guppy_ - Error_RODAN_, thus a positive value means RODAN is more accurate than Guppy. The symbols represent whether the dataset was present ($\bullet$) or absent ($\times$) in the training set of RODAN

While the read accuracies are comparable on most datasets (Supplementary Figure 9), the reads from RODAN have shorter median aligned read lengths in 10 out of 12 datasets (Fig. 5a, Supplementary Table 2). The largest drop in the aligned read lengths is observed in the native SARS2 dataset, where the median read length from RODAN is 708 nucleotides shorter, i.e. a 34% decrease. To explore whether RODAN’s performance is related to read length, we grouped reads by length and found that RODAN performs overall better than Guppy in shorter reads, but the improvement diminishes as read length increases, with the exception of organisms in RODAN’s training dataset (Fig. 5b).

Lastly, the systematic sequencing errors at single nucleotide and motif levels are also prevalent in the reads basecalled by RODAN (Supplementary Figure 10), suggesting that the fundamental causes of sequencing errors reported in this study are not basecaller-specific, but could rather be related to intrinsic difficulties imposed by the raw signal data.

### Update on the direct RNA sequencing kit SQK-RNA004

**Fig. 6:**
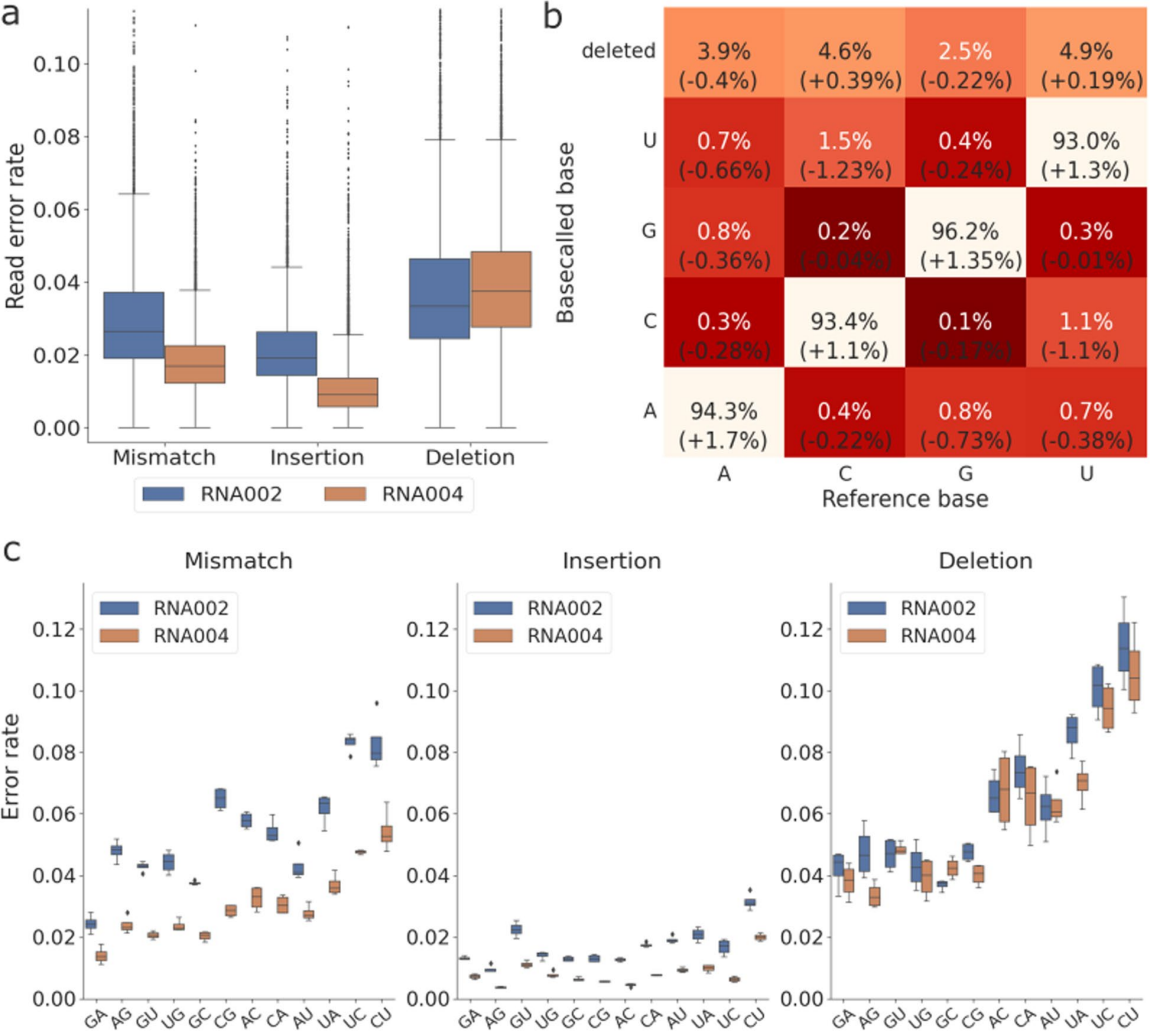
Comparison between the direct RNA sequencing kit SQK-RNA002 and SQK-RNA004. **a** The error rate per read of the curlcake samples, grouped by error type and colored by sequencing kit. **b** Confusion matrix showing the frequencies of each nucleotide being correctly basecalled, miscalled or deleted, based on the curlcakes sequenced with SQK-RNA004. The differences to SQK-RNA002 are shown in the brackets. **c** The mismatch, insertion and deletion rates of 2-mer motifs in the curlcake samples, colored by sequencing kit

To compare the performance of dRNA-seq sequencing kits, we generated synthetic in vitro transcribed “curlcake” RNA with two exisiting direct RNA sequencing protocols, SQK-RNA002 and the latest SQK-RNA004, which was released by ONT at the end of 2023. The curlcake sequences were first introduced in [20] and consist of 4 artificial RNA sequences. Each curlcake sequence is around 2,000 - 3,000 nucleotides in length and was computationally designed to contain all possible 5-mer contexts with high folding energy (software from https://cb.csail.mit.edu/cb/curlcake/). The overall read accuracy of the RNA004 sample is 93.5%, which is an improvement over 92.1% in RNA002. The improvement in read accuracy was mainly a result of reduced mismatch and insertion errors, while deletion error rate remained high (Fig. 6a).

At the single nucleotide level, A had the largest improvement of 1.7% in match rate and a 0.4% decrease in deletion rate with RNA004 (Fig. 6b). Similarly to previous kits, C and U remain the two least accurate nucleotides, with even a slight increase in deletion errors. At the 2-mer level, all motifs saw a decrease in mismatches and insertions, while deletion errors are largely at the same level between the two kits (Fig. 6c). The relative accuracy of 2-mers remain the same as the previous kits, with GA/AG motifs having the least errors and UC/CU having the most, especially with regard to mismatches and deletions.

## Discussion

Since the release of the first MinION device by ONT, the accuracy of nanopore DNA sequencing has significantly improved due to developments in both pore chemistry and basecalling algorithms [3, 33]. At the same time, dRNA-seq has gained considerable popularity due to its reduced bias and the ability to sequence RNA base modifications and characterise the epitranscriptome. However, improvements in the sequencing accuracy of dRNA-seq has so far been limited [5]. Here, we systematically evaluated the accuracy and systematic error patterns of dRNA-seq in datasets covering diverse organisms across the tree of life. We found that the read accuracy is around 90% across organisms in both native and IVT samples, and that deletions account for the majority of errors.

The high error rate of dRNA-seq has several important implications in downstream analyses such as transcript identification and discovery. Firstly, as each native RNA read represents a single polyadenylated RNA transcript that is potentially protein coding, one promising application of dRNA-seq is to identify and quantify the expression of novel transcript isoforms and open reading frames (ORFs). However, the abundance of indel errors can often lead to frameshift errors in gene prediction, a well-known challenge in long-read sequencing [38]. To address this, [11] attempted to reduce errors by correcting dRNA-seq reads with Illumina short reads, yet the remaining indel errors still precluded ORF prediction in more than 80% of the reads.

Another downstream application of dRNA-seq is the detection of RNA base modifications. As nucleotide modifications can alter electric signal and consequently cause basecalling errors, they can be identified through comparison of alignment errors between modified and unmodified control samples [39]. A proportion of the observed errors in our study could be a result of the presence of native base modifications. Notably, the modification-free IVT human dataset has less errors than its native counterpart, but the same pattern is not seen in the two SARS2 datasets. In general, the relationship between errors and modifications is complex: certain types of modifications, such as m^6^A, m^5^C and pseudouridine, can lead to increased basecalling errors, although with strong preferences towards certain motifs [17, 40, 41]. Given that signal deviations arise both from intrinsic errors and modifications, a careful and rigorous investigation is warranted to examine 1) how different modifications can alter signals and 2) how signal changes can affect sequencing errors.

There are generally two sources from which sequencing errors in nanopore sequencing data can arise: firstly, the raw signals produced by the nanopore sequencer and secondly, the basecalling algorithm translating the signal data into nucleotide sequences [33]. In addition to improvements of ONT devices and chemistry for nanopore DNA-seq, there is also continuous development of new basecallers released by both ONT and the broader research community [42, 43]. However, for dRNA-seq data, RODAN [32] remains currently the only published community basecaller outside of ONT. From our evaluation, the performance of RODAN holds up well against Guppy in terms of read accuracy, especially for organisms in its training data. As species-specific training data are known to improve performance of nanopore basecallers in those species [42, 44], the improvements of RODAN suggest a promising direction for training species-specific basecallers also for dRNA-seq data. Lastly, the presence of the same systematic error patterns in RODAN and the latest SQK-RNA004 kit points to more fundamental causes of errors in the raw signal data, necessitating further development of pore chemistry to improve the decoding of raw signal data.

## Conclusions

While dRNA-seq offers exciting opportunities for studying RNA transcription and modifications, our evaluation highlights the need for continued improvements in data quality and accuracy. Clearly, further development and optimization of dRNA-seq protocols, pore chemistry and basecalling algorithms are desirable. With the recent release of the SQK-RNA004 sequencing kit and its improvements in throughput and accuracy, we expect to see more effort in developing this technology and realising its potential. At the same time, appropriate computational methods for data quality control, uncertainty quantification, and error correction are needed to mitigate the effects of high error rates and systematic biases in downstream analyses.

### Supplementary Information

Supplementary Material 1.

## Data Availability

The curlcake datasets with RNA002 and RNA004 kits have been deposited in the European Nucleotide Archive (ENA) at EMBL-EBI under accession number PRJEB73868. The code and scripts to reproduce the analyses and plots are available on https://github.com/KleistLab/nanopore_dRNAseq.
