# Supplementary material for: Sequencing accuracy and systematic errors of nanopore direct RNA sequencing
Source: BMC Genomics. 2024 May 28;25:528. doi: 10.1186/s12864-024-10440-w (PMC11134706; doi:10.1186/s12864-024-10440-w)
Supplement: Supplementary file 1 — Supplementary Material 1. [file 12864_2024_10440_MOESM1_ESM.pdf]

Dataset	Publication	ONT sequencing kit	Reads aligned	med. Q-score	med. length	Acc	Mis	Ins	Del
Human	Jenjaroenpun et al. [2021]	SQK-RNA002	1483755	10.2	876	0.901	0.027	0.026	0.045
Human (IVT)	as above	SQK-RNA002	2015950	10.7	486	0.916	0.022	0.022	0.039
Mouse	Bilska et al. [2020]	SQK-RNA001/2	181977	9.6	616	0.878	0.036	0.025	0.059
Zebrafish	Begik et al. [2022a]	SQK-RNA002	553604	9.6	839	0.867	0.037	0.020	0.073
C. elegans	Roach et al. [2020]	SQK-RNA001	227372	11.0	688	0.915	0.020	0.019	0.044
Arabidopsis	Parker et al. [2020]	SQK-RNA001	1010943	10.7	878	0.911	0.022	0.021	0.045
H. volcanii	Grünberger et al. [2022b]	SQK-RNA001	22315	9.9	505	0.905	0.028	0.022	0.043
E. coli	Grünberger et al. [2022a]	SQK-RNA002	198448	9.3	662	0.876	0.040	0.032	0.049
shortRNAs (IVT)	Begik et al. [2021]	SQK-RNA001	45028	8.5	160	0.899	0.026	0.019	0.051
Yeast	Liu et al. [2019]	SQK-RNA001	324889	9.4	314	0.898	0.024	0.024	0.050
SARS2	Kim et al. [2020]	SQK-RNA002	575673	11.0	2078	0.914	0.022	0.020	0.044
SARS2 (IVT)	as above	SQK-RNA002	2316635	10.5	1572	0.908	0.024	0.023	0.044

Supplementary Table 1: **Details of the datasets.** Statistics regarding the aligned reads (counts, quality scores and lengths) and their accuracies of each dataset included in this study.

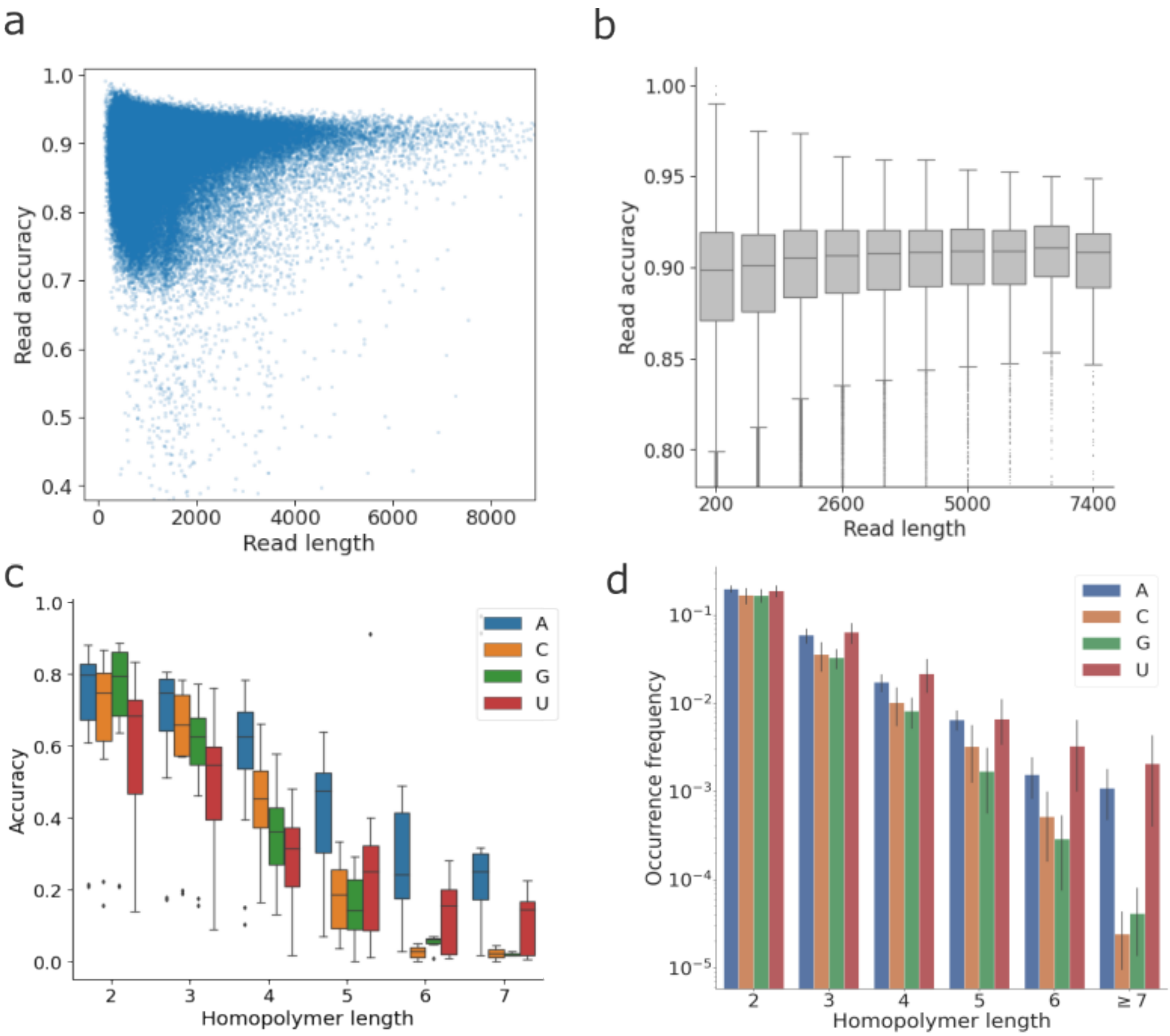

Supplementary Figure 1: a) Scatter plot of read length versus read accuracy, based on the native human dataset. b) A boxplot of the same data, grouped by read length. c) Basecalling accuracy of homopolymers with reference length between 2 and 7, grouped by nucleotides. Each point in a boxplot represents the accuracy of a homopolymer in a dataset, computed by dividing the number of times it is correctly basecalled by its total number of occurrences in the dataset. d) The occurrence frequency distribution of homopolymers at different lengths across all datasets. Each data point is the occurrence frequency of the homopolymer motif in one dataset, normalised by the total number of homopolymers in that dataset.

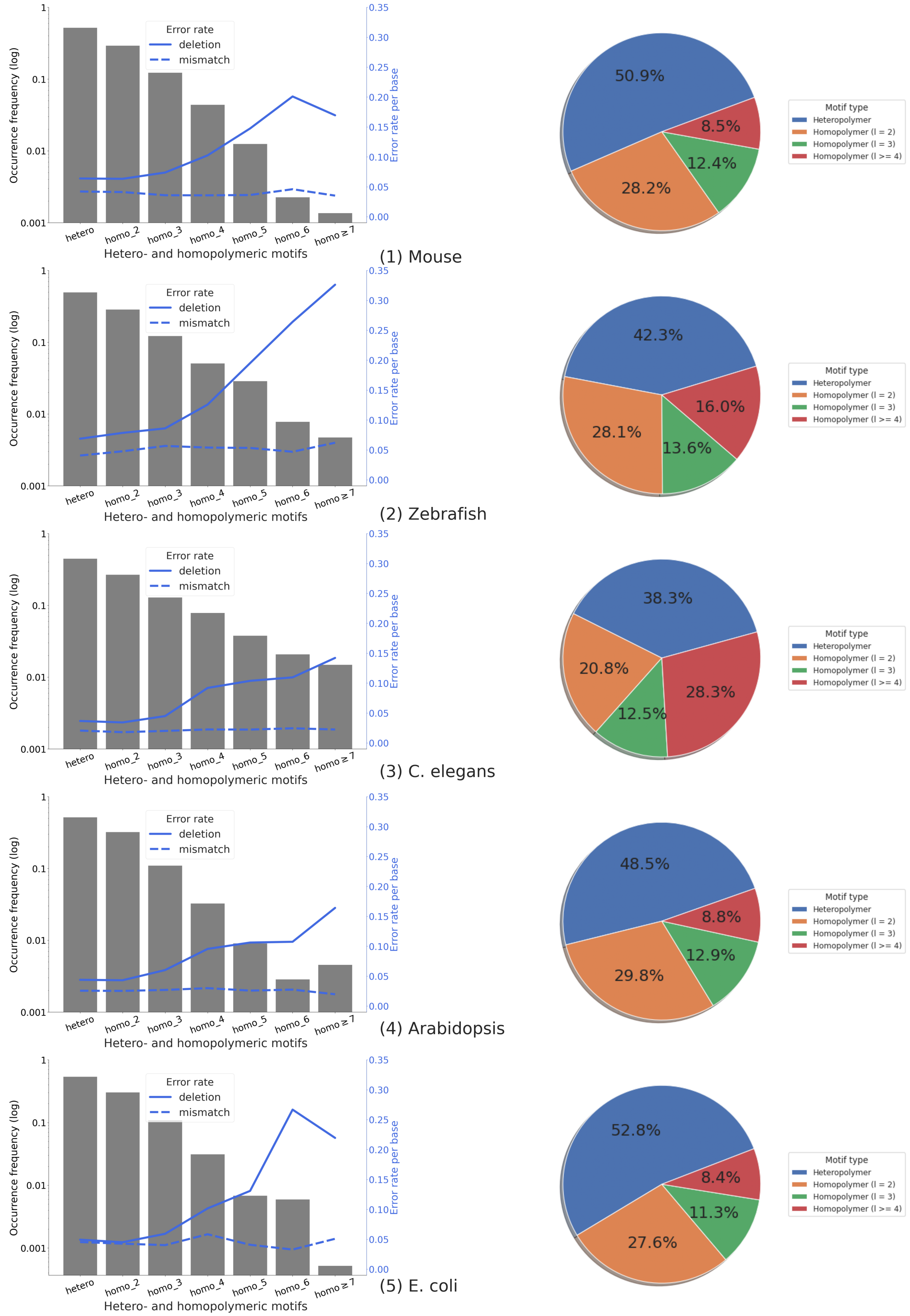

Supplementary Figure 2: **Error distribution in homopolymers versus heteropolymeric regions across species.** Left panel: the occurrence frequency distribution (left axis, in log scale) and the the error rate per base (right axis) in heteropolymeric and homopolymeric motifs of different lengths. Different types of errors are plotted using different line styles. Right panel: the relative distribution of all base errors in homopolymeric and heteropolymeric regions. Homopolymer lengths are indicated in brackets and those of length longer than 3 are grouped together for visualisation.

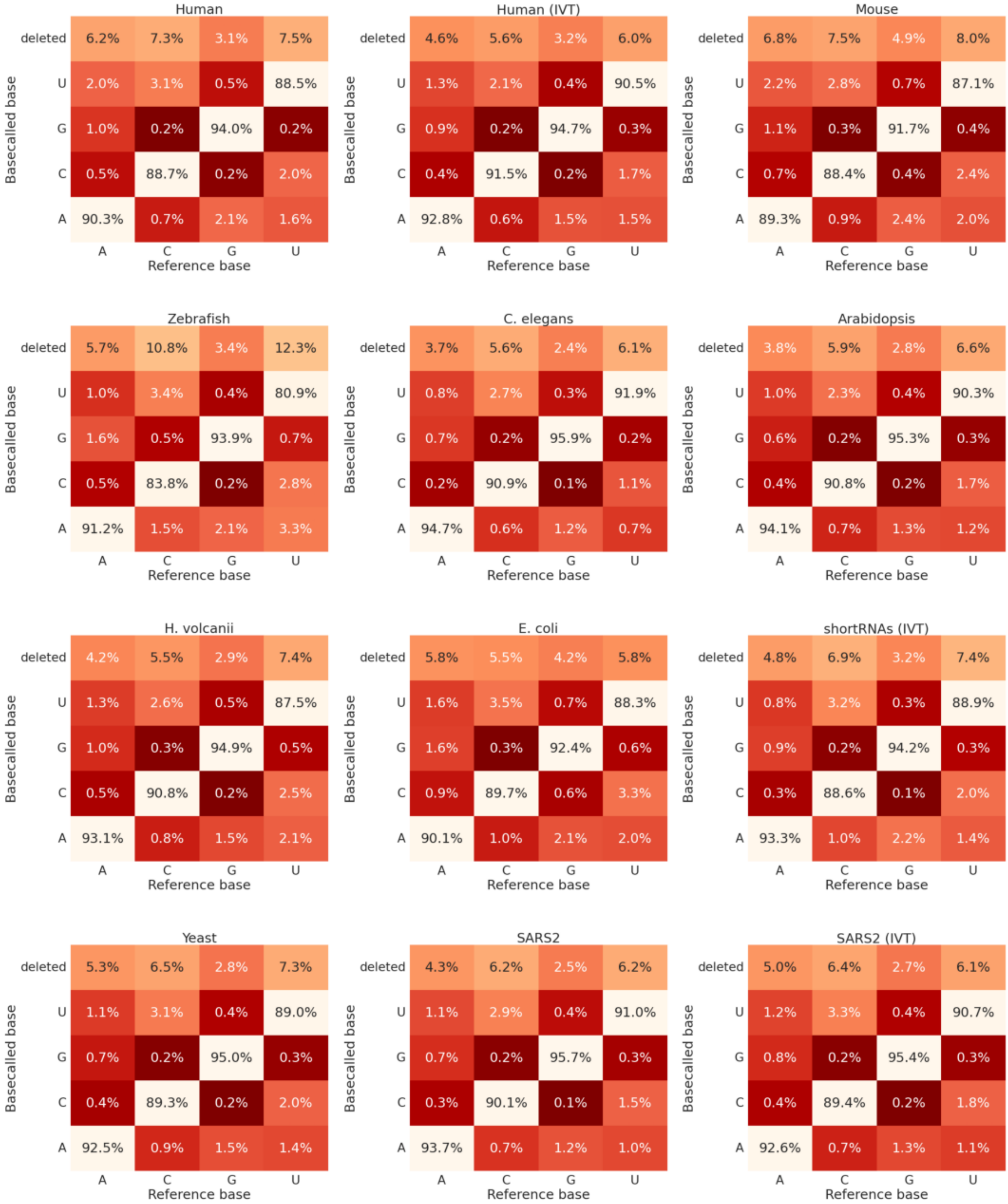

Supplementary Figure 3: **Single nucleotide error profiles across organisms.** Confusion matrix showing the proportion of each base in reference being correctly basecalled, miscalled or deleted, for each organism included in the study.

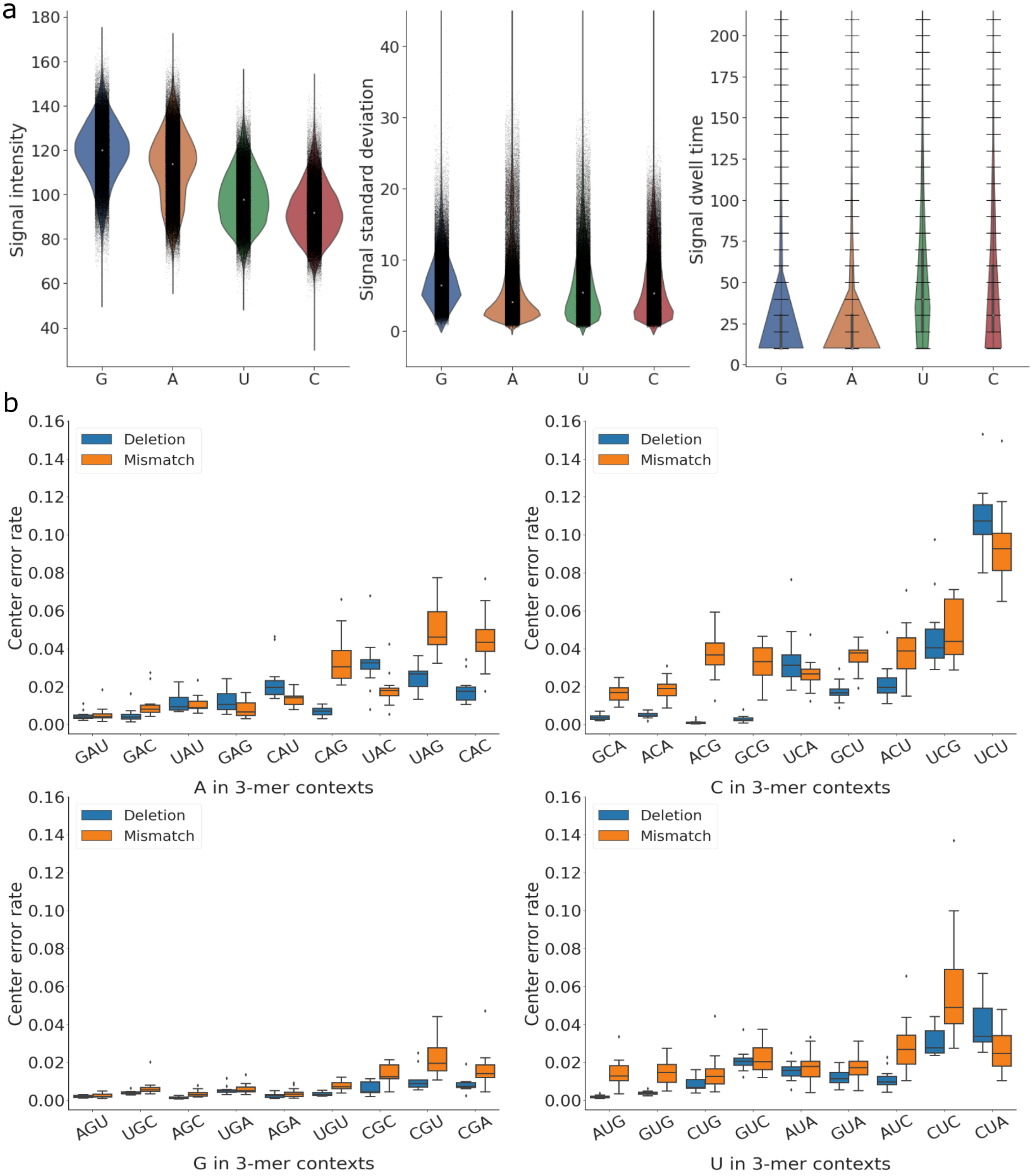

Supplementary Figure 4: a) The distributions of signal features (intensity, standard deviation and dwell time) at correctly basecalled positions, grouped by nucleotide type. b) The error profiles of the center nucleotides across 3-mer contexts, conditional on that the neighboring two bases are correctly basecalled. Each data point represents the mean error rate of all such 3-mer motifs in one dataset.

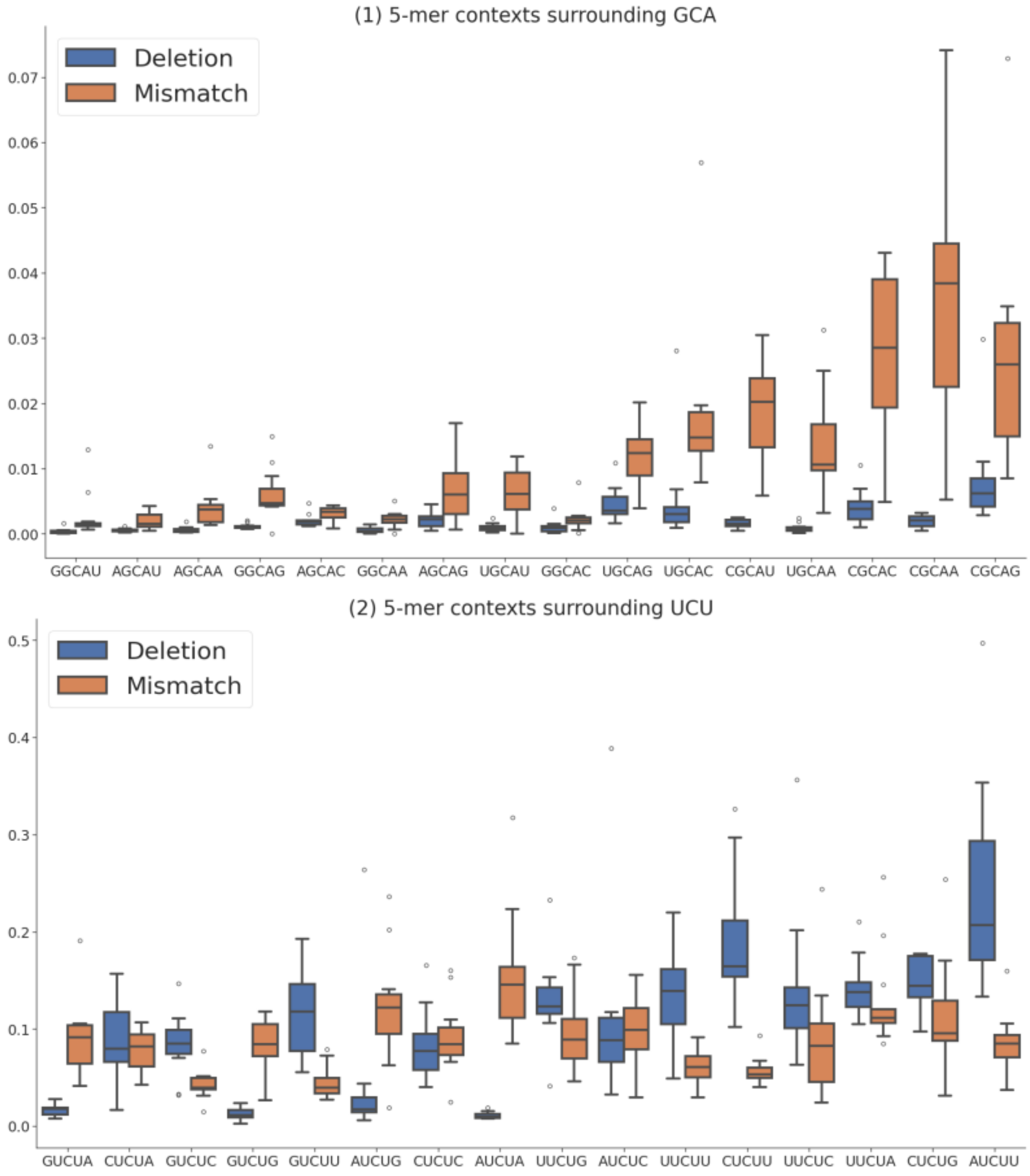

Supplementary Figure 5: The error profiles of the center nucleotides across 5-mer contexts. Here the examples are given where GCA and UCU are the center 3 mer.

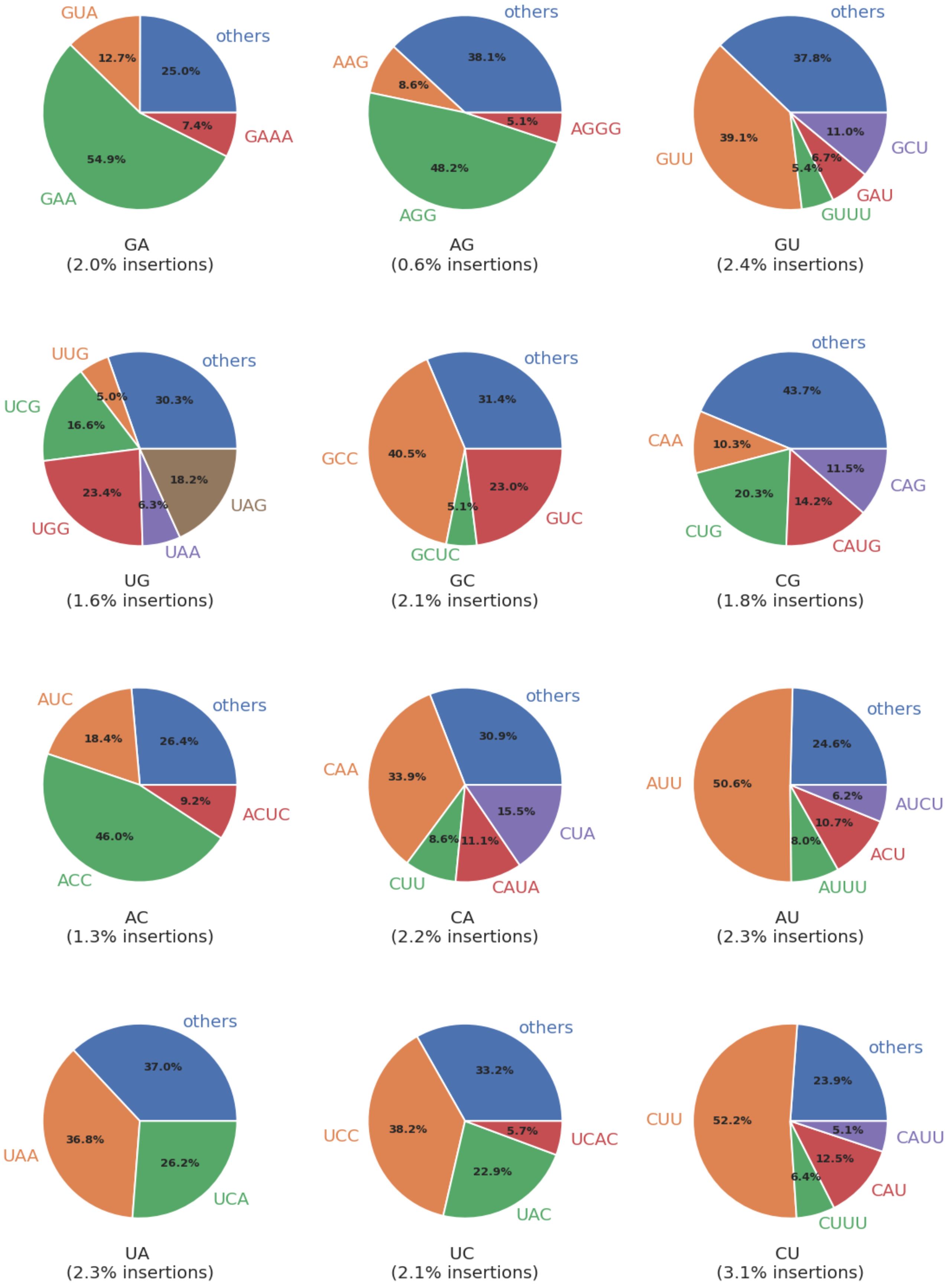

Supplementary Figure 6: **Insertion profiles of 2-mer motifs.** Pie charts showing the proportion of different insertion errors, based on the native human sample. Inserted motifs fewer than 5% of the total number of insertions are grouped into “others”.

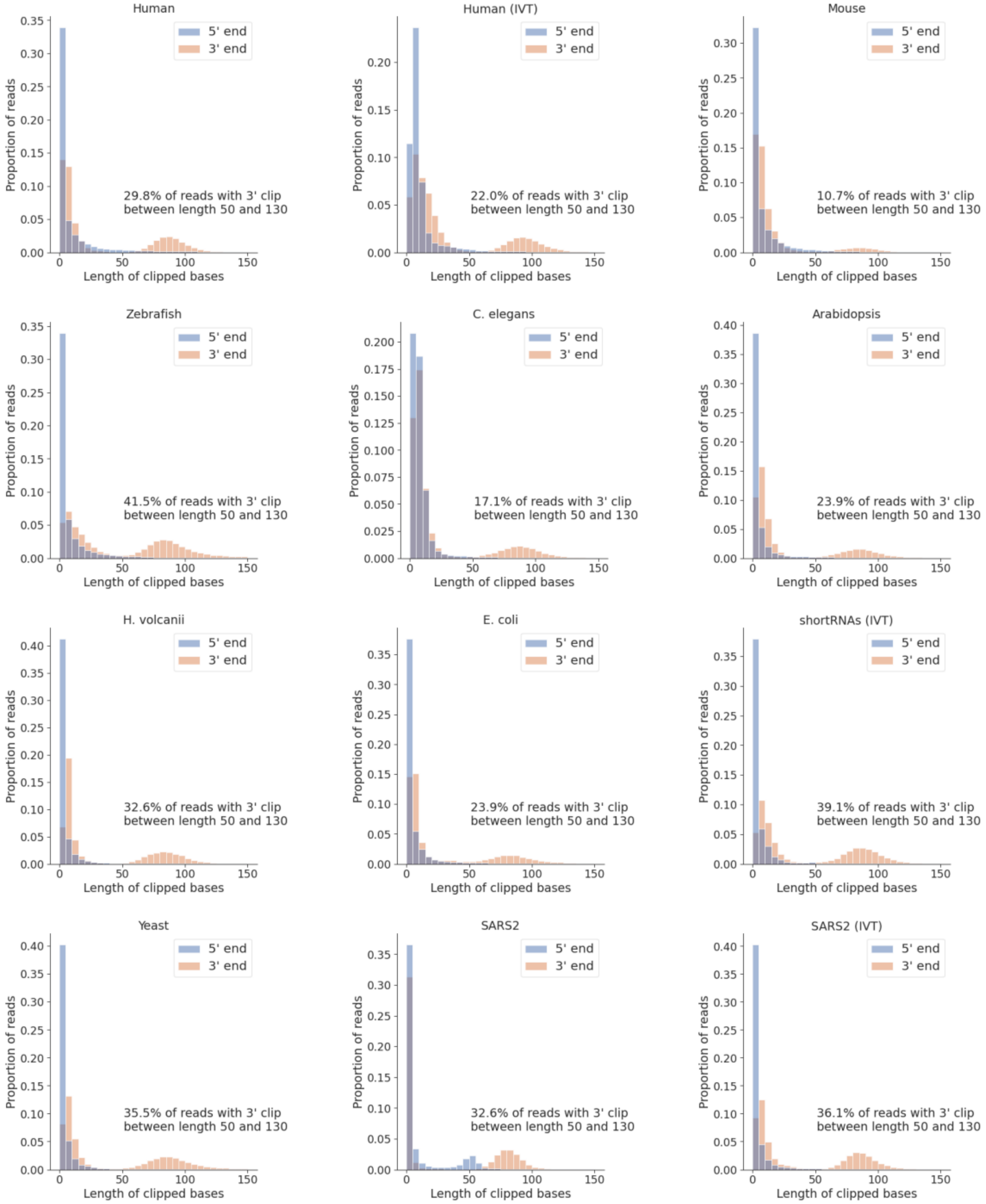

Supplementary Figure 7: **Bias in soft-clipped bases at the 3' end.** The length distribution of the soft-clipped bases at the 5' and 3' ends of reads in each dataset in this study.

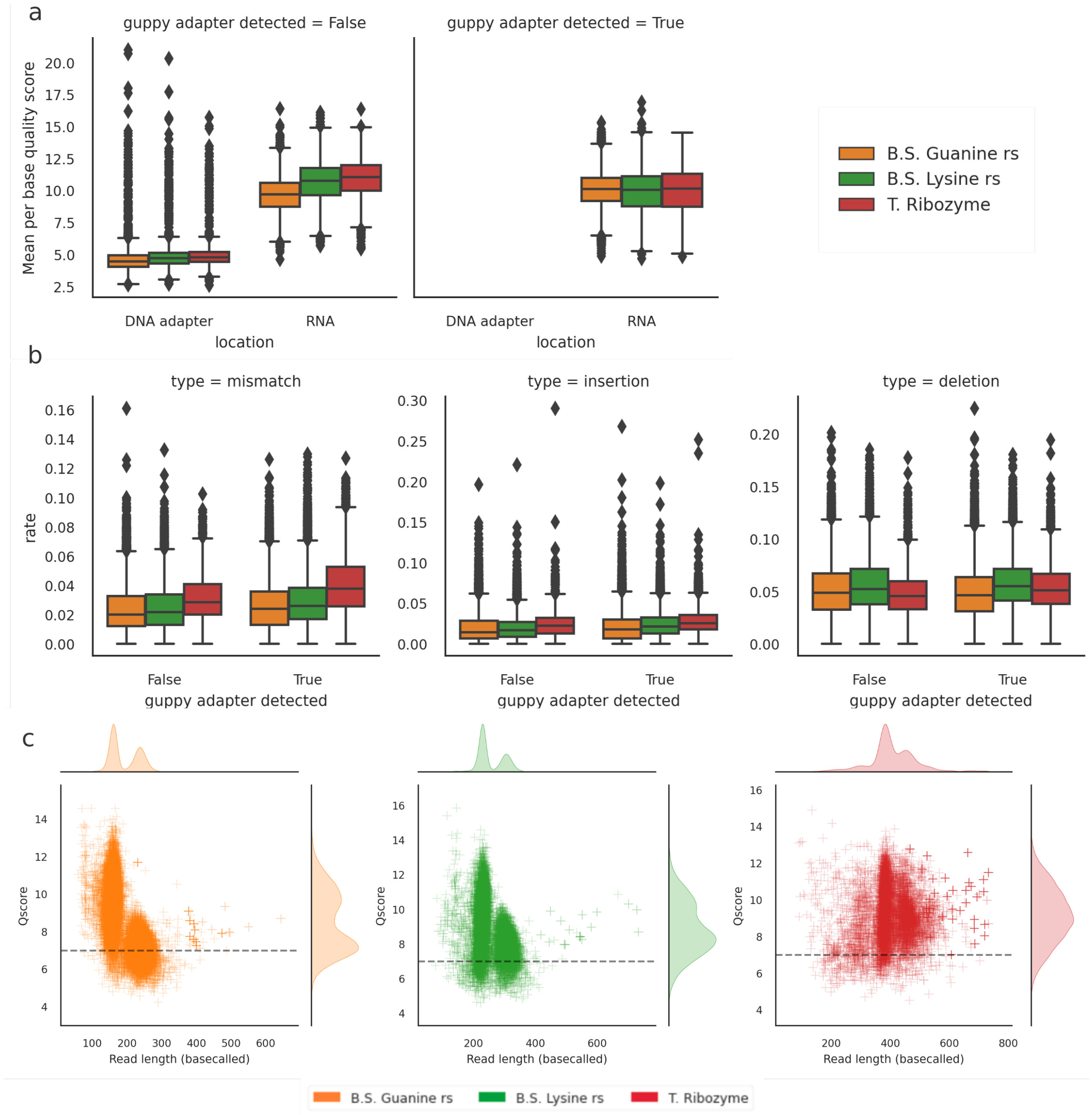

Supplementary Figure 8: **Comparison of reads with and without successful adapter detection.** a) Basecalls in the DNA adapter are of low quality, but adapter detection failure does not appear to affect the quality scores of the subsequent RNA basecalls. b) Failure to detect the adapter does not affect the error rates of the RNA basecalls after alignment. c) The impact of adapter detection failure on basecalled read length and read Q-score in short reads. Adapter detection failure causes bimodality in basecalled read length. The spurious DNA basecalls can significantly impact the read Q-score, potentially leading to data loss. The dashed line represents the conventional filter value of 7. Plots are based on the IVT short RNA dataset.

Dataset	In training data	Reads aligned	med. Length	Acc	Mis	Ins	Del
Human	**yes**	1648051 (+164296)	725 (-151)	0.923 (+0.022)	0.019 (-0.008)	0.021 (-0.004)	0.035 (-0.009)
Human (IVT)	no	1729393 (-286557)	375 (-111)	0.913 (-0.003)	0.023 (+0.001)	0.027 (+0.005)	0.037 (-0.002)
Mouse	no	85286 (-96691)	591 (-25)	0.903 (+0.025)	0.018 (-0.017)	0.015 (-0.01)	0.059 (-0.0)
Zebrafish	no	155285 (-398319)	659 (-180)	0.818 (-0.048)	0.051 (+0.014)	0.018 (-0.002)	0.114 (+0.041)
C. elegans	**yes**	222931 (-4441)	558 (-130)	0.941 (+0.026)	0.012 (-0.008)	0.014 (-0.005)	0.031 (-0.013)
Arabidopsis	**yes**	1097675 (+86732)	471 (-407)	0.903 (-0.008)	0.032 (+0.011)	0.028 (+0.007)	0.033 (-0.012)
H. volcanii	no	14258 (-8057)	959 (+454)	0.916 (+0.011)	0.028 (-0.0)	0.035 (+0.013)	0.021 (-0.022)
E. coli	**yes**	506596 (+308148)	578 (-84)	0.921 (+0.045)	0.025 (-0.015)	0.023 (-0.01)	0.026 (-0.023)
shortRNAs (IVT)	no	41329 (-3699)	170 (+10)	0.919 (+0.02)	0.020 (-0.005)	0.026 (+0.007)	0.027 (-0.024)
Yeast	no	372364 (+47475)	297 (-17)	0.892 (-0.006)	0.028 (+0.004)	0.026 (+0.002)	0.060 (+0.01)
SARS2	no	858189 (+282516)	1370 (-708)	0.916 (+0.002)	0.019 (-0.003)	0.017 (-0.003)	0.041 (-0.003)
SARS2 (IVT)	no	2452303 (+135668)	1103 (-469)	0.920 (+0.012)	0.018 (-0.005)	0.021 (-0.002)	0.033 (-0.011)

Supplementary Table 2: **Basecalling performance of RODAN versus Guppy across organisms.** The differences in each metric from Guppy are indicated in brackets.

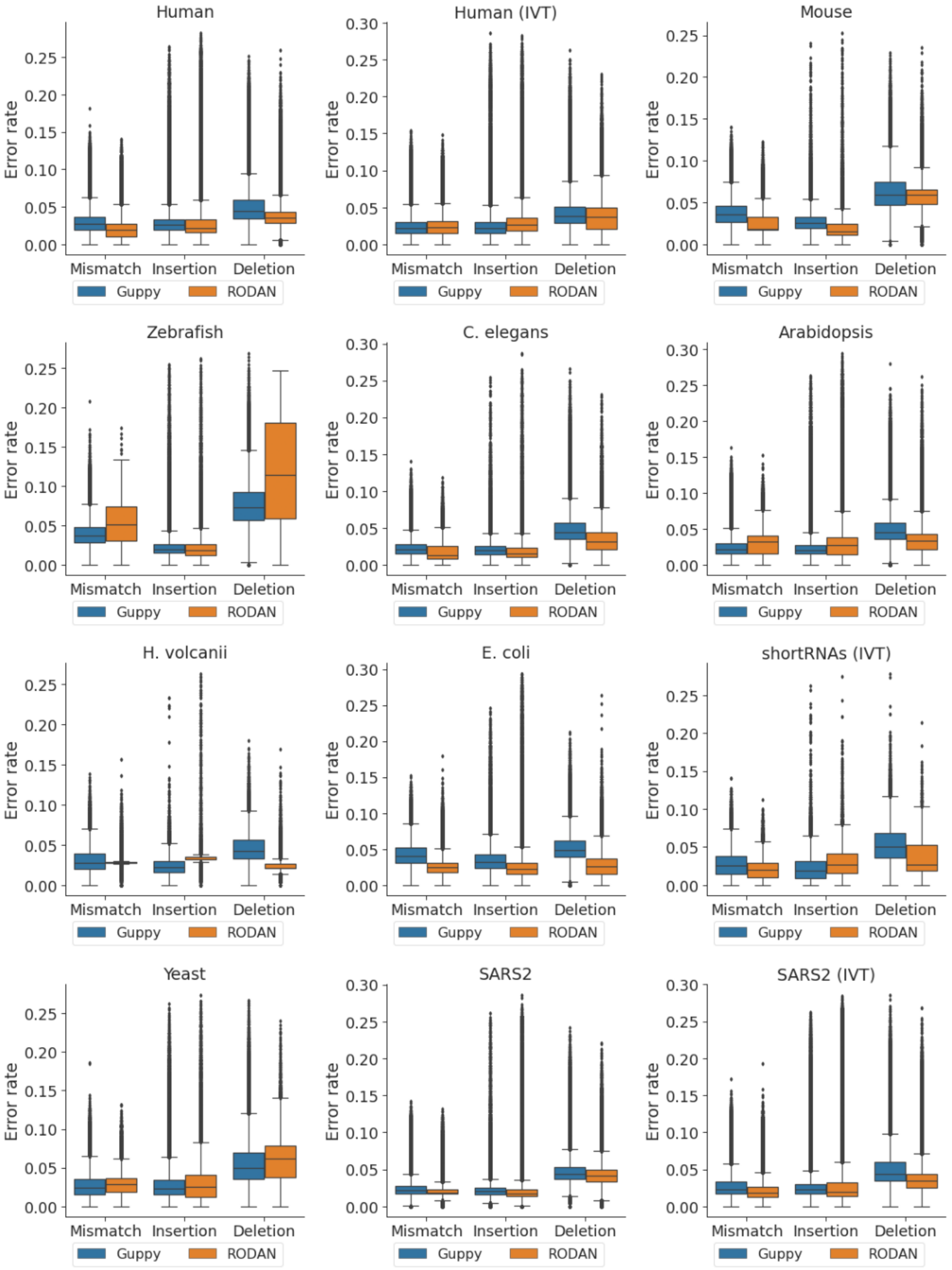

Supplementary Figure 9: **Comparison of basecalling errors between RODAN and Guppy.** The mismatch, insertion and deletion rates for each dataset. Reads with accuracy lower than 70% (or equivalently, more than 30% errors), are filtered for visualization.

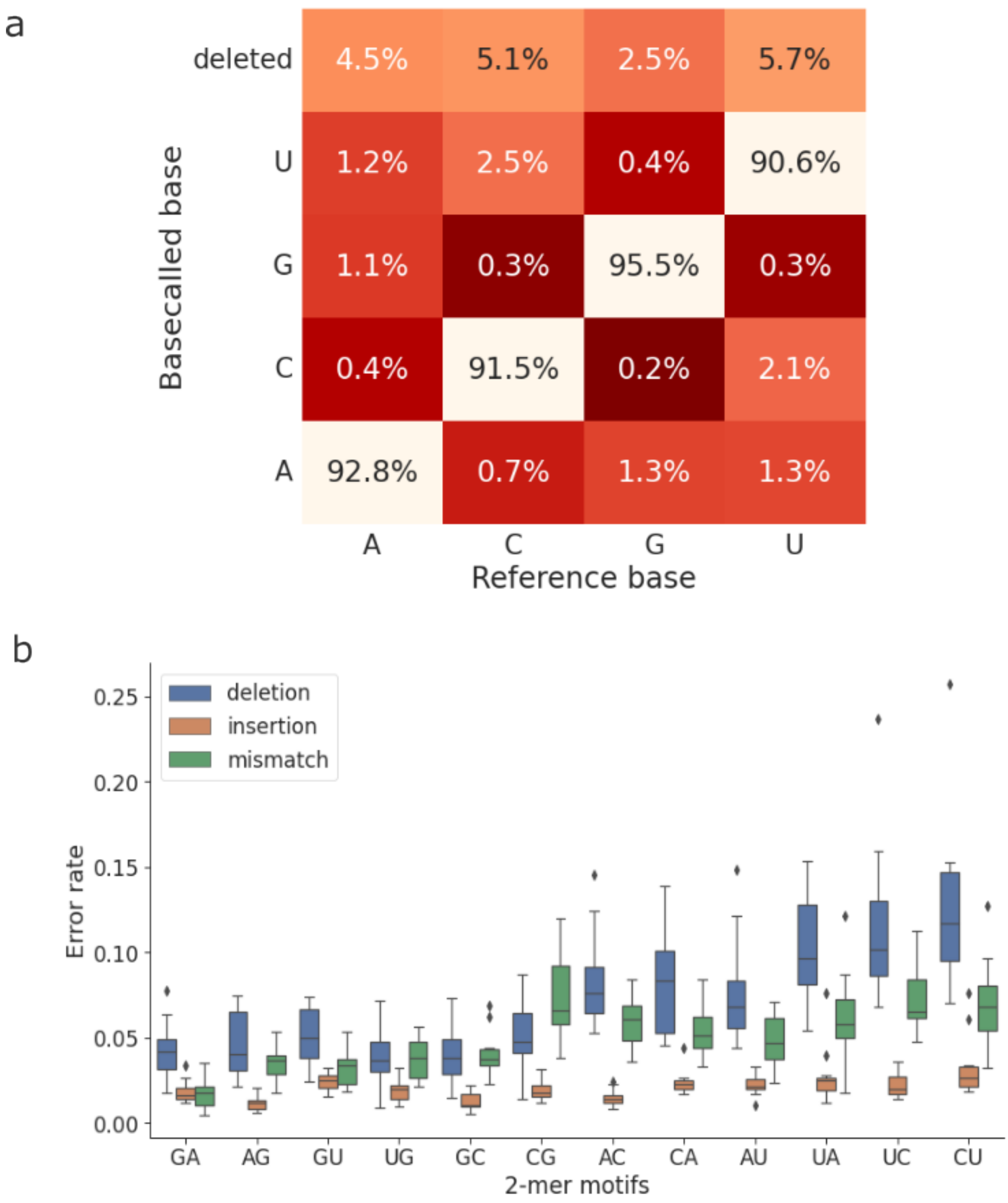

Supplementary Figure 10: **Systematic errors in RODAN.** a) Confusion matrix showing the frequencies of each base being correctly basecalled, miscalled or deleted, computed by taking the mean of all samples basecalled by RODAN. b) The error rate of 2-mer motifs across the samples basecalled by RODAN, grouped by error type. Each data point corresponds to the mean error rate of the motif in one dataset.
